# A Genomic Analysis of Factors Driving lincRNA Diversification: Lessons from Plants

**DOI:** 10.1534/g3.116.030338

**Published:** 2016-07-15

**Authors:** Andrew D. L. Nelson, Evan S. Forsythe, Upendra K. Devisetty, David S. Clausen, Asher K. Haug-Batzell, Ari M. R. Meldrum, Michael R. Frank, Eric Lyons, Mark A. Beilstein

**Affiliations:** * School of Plant Sciences, University of Arizona, Tucson, Arizona 85721; † Department of Forest Ecosystems and Society, Oregon State University, Corvalis, Oregon 97331; ‡ Department of Statistics, University of Washington, Seattle, Washington 98195; § Genetics Graduate Interdisciplinary Group, University of Arizona, Tucson, Arizona 85721

**Keywords:** lincRNA, Brassicaceae, comparative genomics, evolution, transcriptomics

## Abstract

Transcriptomic analyses from across eukaryotes indicate that most of the genome is transcribed at some point in the developmental trajectory of an organism. One class of these transcripts is termed long intergenic noncoding RNAs (lincRNAs). Recently, attention has focused on understanding the evolutionary dynamics of lincRNAs, particularly their conservation within genomes. Here, we take a comparative genomic and phylogenetic approach to uncover factors influencing lincRNA emergence and persistence in the plant family Brassicaceae, to which *Arabidopsis thaliana* belongs. We searched 10 genomes across the family for evidence of > 5000 lincRNA loci from *A. thaliana*. From loci conserved in the genomes of multiple species, we built alignments and inferred phylogeny. We then used gene tree/species tree reconciliation to examine the duplication history and timing of emergence of these loci. Emergence of lincRNA loci appears to be linked to local duplication events, but, surprisingly, not whole genome duplication events (WGD), or transposable elements. Interestingly, WGD events are associated with the loss of loci for species having undergone relatively recent polyploidy. Lastly, we identify 1180 loci of the 6480 previously annotated *A. thaliana* lincRNAs (18%) with elevated levels of conservation. These conserved lincRNAs show higher expression, and are enriched for stress-responsiveness and *cis*-regulatory motifs known as conserved noncoding sequences (CNSs). These data highlight potential functional pathways and suggest that CNSs may regulate neighboring genes at both the genomic and transcriptomic level. In sum, we provide insight into processes that may influence lincRNA diversification by providing an evolutionary context for previously annotated lincRNAs.

Long noncoding RNAs (lncRNAs) are defined as transcripts that are > 200 nt in length but are not predicted to encode polypeptides of > 100 amino acids (Liu *et al.* 2012). Reported lncRNA repertoires in mammals vary, but are commonly in the thousands to tens of thousands of transcripts, accounting for ∼90% of the genome (Derrien *et al.* 2012; Cabili *et al.* 2011; Stamatoyannopoulos *et al.* 2012). The biological roles of a few lncRNAs, such as the telomerase RNA (TER), COOLAIR, Xist, and MALAT1 are well characterized (Blackburn and Collins 2011; Pontier and Gribnau 2011; Gutschner *et al.* 2013). These RNAs function in genome maintenance, chromosome silencing, stress response, and alternative splicing, respectively. Despite these key examples and the prevalence of lncRNAs within genomes, functional data for the majority of lncRNAs are lacking.

Much of what we know about lncRNAs is derived from extensive next-generation sequencing in mammalian systems. On average, mammalian lncRNAs are transcribed at ∼10-fold lower levels than protein-coding genes (Cabili *et al.* 2011; Managadze *et al.* 2011). In addition, a majority of lncRNAs in mice and humans are tissue specific, with many lncRNAs restricted to the brain, liver, or testes (Necsulea *et al.* 2014). LncRNAs are processed similarly to mRNAs: they are transcribed predominantly by Pol II, capped, polyadenylated, and composed of multiple exons (Ponting *et al.* 2009). Moreover, lncRNA loci exhibit epigenetic marks associated with active chromatin (Cabili *et al.* 2011).

lncRNAs are often categorized based on the genomic context from which they are transcribed. Some lncRNAs are embedded within, or overlap with, protein-coding genes (Ponting *et al.* 2009). These lncRNAs are further classified into different categories based on directionality of overlap, and the degree to which transcription varies from the related protein-coding gene. Overlapping lncRNAs can serve as key regulators of the genes to which they are linked (Wang and Chang 2011). For example, a subset of lncRNAs that overlap a protein-coding gene in the antisense direction function as *cis*-natural antisense transcripts (*cis*-NATs) (Lapidot and Pilpel 2006). A specific subgroup of lncRNAs originate in intergenic regions, and are referred to as long intergenic noncoding RNAs (lincRNAs). LincRNAs are autonomous transcriptional units, in that their transcription does not appear to be dependent on that of adjacent genes (Cabili *et al.* 2011), and thus these molecules may function in molecular pathways independent of neighboring genes (Ulitsky and Bartel 2013). Categorizing lincRNAs based on functional characteristics remains a challenge. We will focus specifically on the intergenic class of lncRNAs in this manuscript.

Recent comparative analyses in mammals have demonstrated that lncRNA populations display poor genomic and transcriptomic conservation relative to protein-coding genes (Necsulea *et al.* 2014; Hezroni *et al.* 2015). Lack of conservation is derived in part from relaxation of constraint on nucleotide evolution (Ponjavic *et al.* 2007). A relatively large proportion of lncRNAs are species-specific (Hezroni *et al.* 2015), suggesting lack of constraint on nucleotide evolution is not the only factor leading to diversification. However, the factors affecting the emergence of new lncRNAs are not well understood.

While the origins of most lncRNAs are unknown, three scenarios have been proposed for emergence of new lncRNA loci (Ulitsky and Bartel 2013; Ponting *et al.* 2009): pseudogenization, gene duplication, or *de novo* transcription from a previously silent locus. Although they make up a small portion of the overall number of mammalian lncRNAs, there is ample evidence for the role of pseudogenization in the emergence of lncRNAs (Ulitsky and Bartel 2013). Pseudogenized loci often remain transcriptionally active, albeit at lower levels, and are, by definition, noncoding (Pink *et al.* 2011). The role of gene duplication in lncRNA emergence is less clear. Most lncRNAs appear to be single copy in vertebrates, but these inferences are based on presence or absence of similar sequences among related species (Ulitsky *et al.* 2011), rather than using a phylogenetic approach to infer duplication history. Most lncRNAs appear to emerge *de novo*, and transposable elements (TEs) may play a key role in this emergence. Compared with protein-coding genes, TE-derived repetitive sequences are more prevalent in mammalian lncRNAs; they account for 30% of total lncRNA sequence in humans (Kapusta *et al.* 2013). While there is evidence to suggest that TEs contribute to sequence diversification of lncRNA loci, it is unclear if TEs drive the emergence of novel lncRNAs.

A subset of lncRNAs display lower rates of evolution, presumably due to conservation of function. Examples of conservation of synteny, sequence, structure, or gene organization are seen in the lncRNAs TER, Xist, and COOLAIR (Wang and Chang 2011; Ulitsky and Bartel 2013; Castaings *et al.* 2014). The telomerase RNA, TER, an essential lncRNA that participates in genome maintenance, displays conservation of sequence and synteny within major eukaryotic clades, and major structural elements tied to function are conserved among fungi, ciliates, and vertebrates (Xiaodong Qi *et al.* 2013; Chen *et al.* 2000). Xist is a eutherian lncRNA that is responsible for X-chromosome inactivation. A lncRNA with overall poor sequence conservation, *Xist* loci are conserved syntenically in eutherians in functional repeat units (Elisaphenko *et al.* 2008; Duret *et al.* 2006; Romito and Rougeulle 2011). COOLAIR is a lncRNA involved in regulating flowering in response to temperature in the plant family Brassicaceae (Castaings *et al.* 2014). COOLAIR is syntenic within sampled Brassicaceae, and functionally important domains are conserved. Thus, as with protein-coding genes, function likely constrains sequence and positional evolution for a subset of lncRNAs.

In plants, lincRNA datasets have been inferred from transcriptome data for *Arabidopsis thaliana*, *Populus trichocarpa*, and *Zea mays*, among others (Liu *et al.* 2012; Shuai *et al.* 2014; Li *et al.* 2014). The most comprehensive lincRNA annotation exists for *A. thaliana*, where a detailed analysis of 200 tiling arrays and numerous RNA-seq datasets uncovered 13,230 intergenic transcripts, of which 6480 were classified as lincRNAs (Liu *et al.* 2012). Similar to their mammalian counterparts, *A. thaliana* lincRNAs (AtlincRNAs) are processed like mRNAs, expressed at low levels, and a subset display tissue-specificity. Homology searches in poplar and grape yielded hits for < 1% of AtlincRNAs, suggesting they may be conserved at lower rates than mammals. In plants, genomes separated by ≥ 100 million yr of evolution [for example, Arabidopsis and poplar diverged ∼100 million yr ago (Mya) (Magallón *et al.* 2015)], appear unlikely to yield comparative data useful for distinguishing between conserved and species-specific lincRNAs. Fortunately, *A. thaliana* is a member of the plant family Brassicaceae, which arose ∼54 Mya (Beilstein *et al.* 2010), and for which a wealth of genomic and transcriptomic data are publicly available. As a result, the family is ideal for evolutionary comparisons, and thus provides a framework to infer factors influencing lnc/lincRNA diversification more broadly.

We present an evolutionary and comparative genomic analysis of > 5000 lincRNAs in *A. thaliana* and its relatives within Brassicaceae spanning 54 million yr of divergence. For our comparative analyses, we used genome data from 10 species within the Brassicaceae plus *Tarenaya hassleriana*, a member of the sister lineage Cleomaceae (Figure 1) (Beilstein *et al.* 2006; Cheng *et al.* 2013; Hall *et al.* 2002). Other studies have used lnc/lincRNAs as characters projected at the tips of an organismal tree (Necsulea *et al.* 2014; Hezroni *et al.* 2015). While trees used in this way are powerful tools for inferring evolutionary patterns, here we take an explicitly phylogenetic approach to understand the dynamics of lincRNA evolution. Using sequence similarity, we reconstructed families of homologous lincRNA loci, aligned the constituent sequences, built gene trees, and used gene tree/species tree reconciliation to infer evolutionary processes. The advantage of this method is that it allows us to investigate factors affecting lincRNA emergence and decay. Our results indicate that small-scale duplication events impact lincRNA emergence more than whole genome duplication (WGD) events or activity of TEs. WGD events appear to have propelled the loss of putative lincRNA loci relative to protein-coding genes. In addition, we identified a subset of AtlincRNAs that are conserved across the sampled Brassicaceae genomes. These conserved AtlincRNAs are more likely to be stress-responsive and enriched for *cis*-regulatory elements, suggestive of both a function, and a reason for conservation.

**Figure 1:**
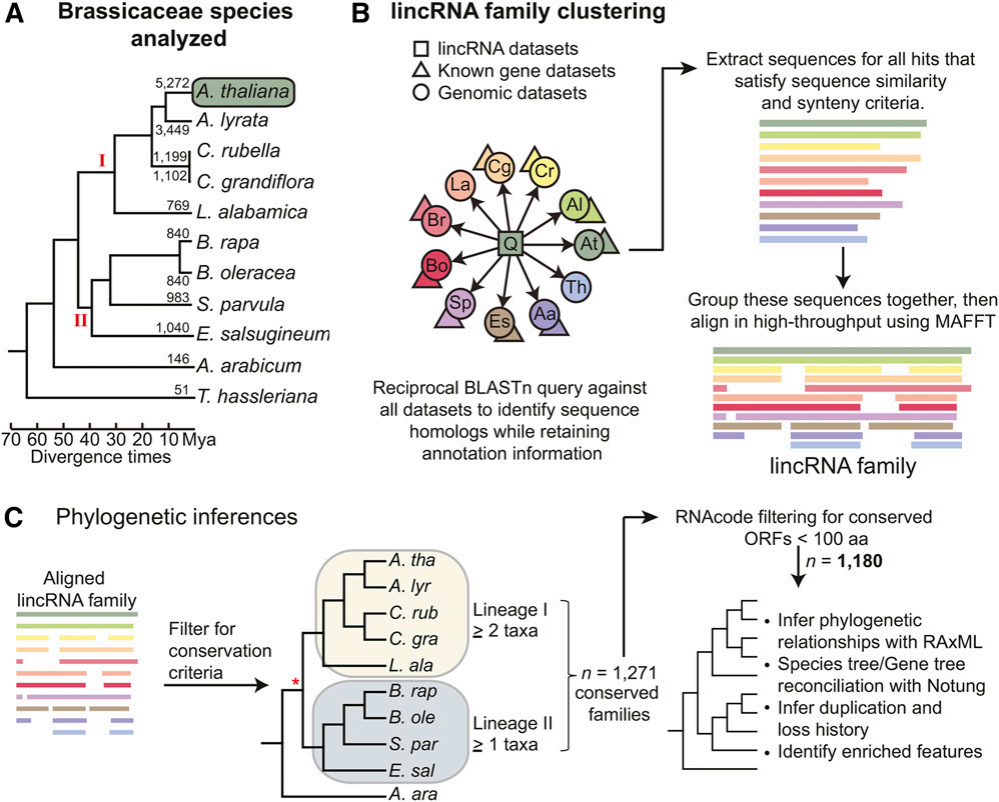
Schematic representation for identification, clustering, and phylogenetic analysis of AtlincRNAs and their homologous loci. (A) Species analyzed within Brassicaceae. A chronogram of the Brassicaceae species, and outgroup *T. hassleriana*, used in this study. Lineages I and II are indicated in red. Number of homologous AtlincRNA loci detected in each species shown. (B) General scheme for identifying AtlincRNA sequence homologs in other species. The Liu *et al.* (2012) lincRNA dataset (dark green box denoted by “Q”) were used as the query in a reciprocal BLAST of genomes (round colored circles). Overlap between identified sequence homologs and known gene datasets (colored triangles) was determined for annotation purposes. Homologous sequences, along with available annotations, were extracted and aligned. Colored lines represent sequences, and are color coded to match the genomes from which they were extracted. (C) Phylogenetic inferences of conserved AtlincRNA families. Currently accepted species relationships, with two lineages, indicated (center). A red asterisk represents the last common ancestor of lineage I and II species. Aligned AtlincRNA families were filtered according to the conservation criteria shown. The number of conserved AtlincRNA families (all species combined) passing through each phylogenetic analysis step is listed.

## Materials and Methods

### Identification of orthologous AtlincRNA loci in Brassicaceae

AtlincRNAs were used as a query in a BLAST (Altschul *et al.* 1990) against the genomes of 10 Brassicaceae and one outgroup (*T. hassleriana*), using the following parameters: (penalty –2, reward 1, gapopen 5, gapextend 2, wordsize 8, evalue 1e–20). All genomes are listed in Supplemental Material, File S1. Close hits (those closer together than the original size of the query lncRNA) were merged, top blast hits from each species designated, and then FASTA sequences extracted for each hit. The adjacent protein-coding genes on either side of the lincRNA (or a 5 kb region if protein-coding genes were lacking) were used in a separate series of reciprocal BLASTs to determine if the top lincRNA hits from each species were syntenic as well as sequence similar. Only the top BLAST hit for each lincRNA in each genome was analyzed for synteny. In addition, the top BLAST hit was used as query in a separate reciprocal BLAST against the original query genome to determine reciprocity. Only top BLAST hits that were syntenic and reciprocal were denoted as sequence homologs. Each sequence name includes subject species name, query lncRNA name, and species, followed by a unique identifier. Any hits that overlapped with a known gene had that gene ID appended to their ID. Sequences for each hit were extracted from the appropriate genome and clustered together into a family with an ID corresponding to the query. Similar parameters were used with a dataset of 10,000 human lncRNAs from the LNCipedia.org (Volders *et al.* 2013) dataset (version 3.1) for identifying orthologous loci in the genomes of chimp, orangutan, and mouse. Alignments were performed using MAFFT (Katoh and Standley 2013) from the command-line using standard parameters. These alignments were used for downstream phylogenetic analyses. RNAcode (Washietl *et al.* 2011) was performed using standard parameters on alignments that contained at least four taxa. All lincRNA families identified with a query lincRNA containing a small ORF were removed from our analysis.

### Calculating transposable element content in lncRNAs

To determine TE content in lncRNAs, we masked the Arabidopsis genome using RepeatMasker (Smit *et al.* 2015). The Arabidopsis repeat database was acquired from RepBase (Genetic Information Research Institute). In addition to sequences present in RepBase, we added sequence for known transposable elements found in the TAIR10 annotation. RepeatMasker, and all dependencies were run according to parameters previously used in mammals (Kapusta *et al.* 2013). Exonic and intronic sequence was used for calculating TE overlap with protein-coding and lincRNA loci, with the exception of 5′ and 3′ UTRs for protein-coding genes.

### Conservation of expression and structure

Correlating conservation to RNA-seq FPKM (fragments per kilobase of transcript per million mapped reads) values was performed using values reported by Liu *et al.* (2012). *A. thaliana* lincRNAs were binned according to the phylogenetic depth to which they were conserved in the family. Minimum free energies were calculated for each of these lincRNAs using RNAfold (Vienna Package 2.0; Lorenz *et al.* 2011) in high-throughput. As these lincRNAs varied in length substantially, for direct comparison, an average MFE was calculated by dividing the MFE by the length of the lncRNA.

### Inferring lincRNA loss and decay

For the conserved AtlincRNA families with missing loci in another species, reciprocal BLASTN was rerun on this species’ genome using a less stringent 1e–5 cutoff value. Additionally, BLASTN was performed in the same genome using protein-coding genes adjacent to the AtlincRNA using the 1e–5 value. This lower E-value was used to account for potential decay of adjacent protein-coding genes, such as in the mesopolyploid species. Genomic coordinates for all lincRNA BLAST returns at the 10^−5^ threshold were compared to the coordinates for returns of the BLAST of the *A. thaliana* adjacent protein-coding genes. For a lincRNA BLAST return to be considered a homologous locus undergoing sequence decay, the two regions must fall within 10 kb of one another on the same chromosome (or greater if the nearest protein-coding gene was further away from the AtlincRNA). BLAST returns that did not meet this criteria, or absence of any lincRNA BLAST return at the lower threshold were considered loss events. Loss was confirmed using the comparative genomics platform CoGe (Lyons *et al.* 2008; https://genomevolution.org/CoGe/). Loss *vs.* decay of protein-coding genes was inferred in a similar manner.

### Inferring dating of duplication events

Maximum likelihood phylogenetic trees were inferred from each nucleic acid alignment with RAxML version 7.2.8 (Stamatakis 2014) using a general time reversible (GTR) model with gamma distributed rate heterogeneity. Support values were calculated from 100 bootstrap replicates. The topology of each gene tree was reconciled to the known species topology using Notung version 2.6 (Durand *et al.* 2006). Trees were rooted in Notung using the *root* function, which roots each along the branch that provides the most congruence with the species tree. The *rearrange* function was used to rearrange poorly supported (<70% bootstrap support) relationships to reflect the species topology. Inferred duplication information was extracted from Notung output *info* files, and *png* files were generated for visual inspection and downstream analysis.

### Inferring characteristics that correlate with conservation

To identify AtlincRNA families with miRNA binding motifs, we ran all the sequences from each family through the miRNA prediction software psRNATarget (http://plantgrn.noble.org/psRNATarget/) (Dai and Zhao 2011) using only miRNAs identified in Brassicaceae. For psRNATarget, more stringent cut-off threshold of 2.0 was used, with the length for complementarity scoring set at 20 nt, with the flanking region around the target set at 17 bp upstream and 13 bp downstream (standard settings). Stress-responsive lincRNAs were identified from the Liu *et al.* (2012) dataset. The genomic locations of conserved noncoding regions were obtained from Haudry *et al.* (2013). Bedtools overlap was used to determine if lincRNA loci overlapped with these CNS (Quinlan and Hall 2010).

### Statistical analyses

**Figure 2:**
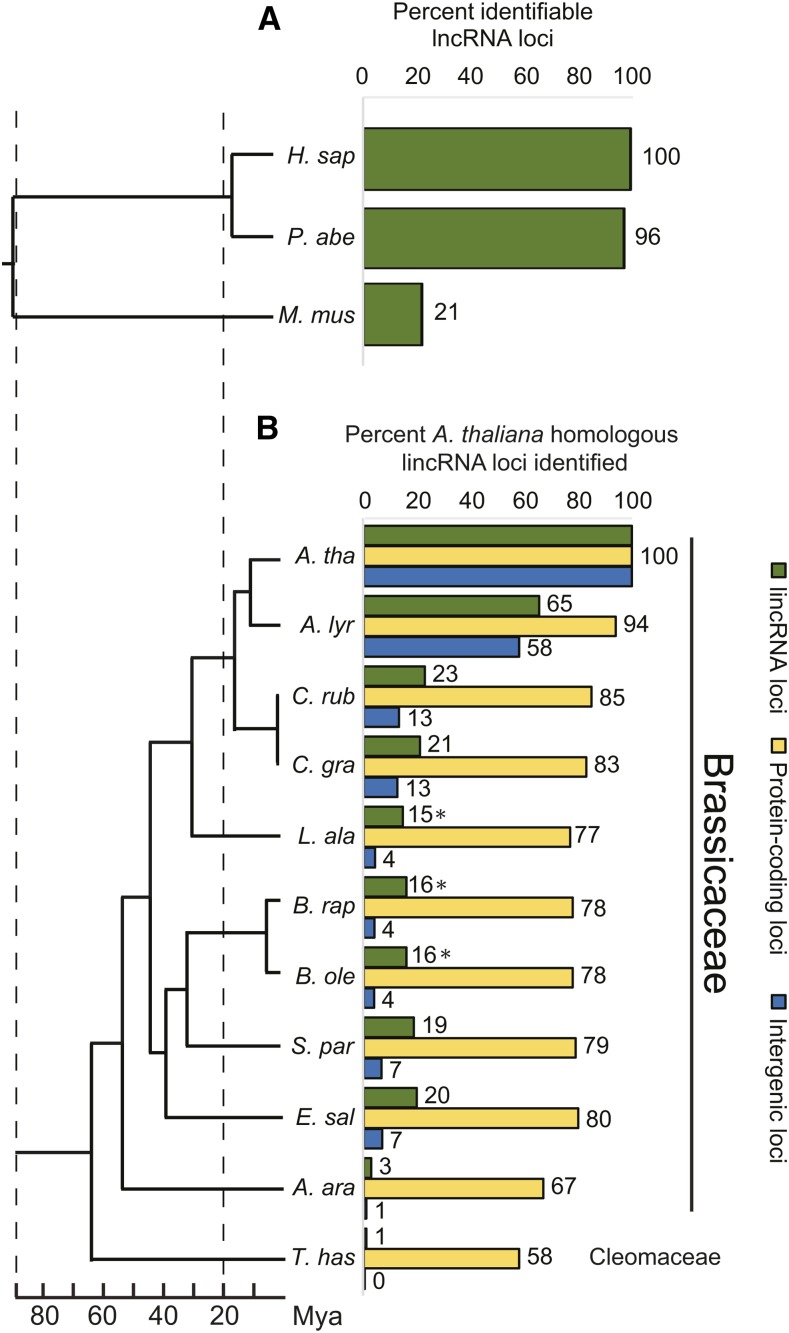
Comparison of conserved homologous lincRNA loci in select mammals and Brassicaceae. (A) Percent of human (*H. sap*) lncRNA homologs identified in close relatives. Percentage of recovered loci are shown next to each bar. The accepted organismal phylogeny and estimated times of divergence for these three species was derived from Arnason *et al.* (2008) and is shown to the left to allow for direct comparisons. (B) Percentage of homologous loci recovered for AtlincRNAs (green), genic (yellow) and intergenic (blue) loci using a similar search protocol as that shown for human lncRNAs. Species’ names are abbreviations of those shown in Figure 1A. Percentage is shown next to each bar. Divergence times and species phylogeny was obtained from Beilstein *et al.* (2010).

Fisher’s exact test was used when comparing the observed numbers of identified lincRNAs in Figure 2. In each case, the observed lincRNAs in the indicated species (either *Leavenworthia alabamica*, *Brassica rapa*, or *Brassica oleracea*) were compared to an expected value based on the number of observed instances in equally or more divergent species (the average of *Eutrema salsugineum* and *Schrenkiella parvula* were used in both cases). When comparing the correlation between conservation of AtlincRNAs *vs.* expression of that locus in *A. thaliana*, we used a linear regression analysis to identify the significance, and established a Pearson’s correlation coefficient. A score test was performed with a Bonferroni multiple comparison correction for the lincRNA loci loss and decay analysis. Score intervals and score tests are reported in File S2.

### Data availability

The datasets used in this study were acquired from publically available resources and are listed in File S1. Additional information pertaining to analyses is available upon request.

## Results

### AtlincRNA loci are conserved at an intermediate level when compared to protein-coding genes or intergenic regions

To characterize the evolution of plant lincRNAs, we focused on a recently published dataset of ∼6500 Arabidopsis lincRNAs (Liu *et al.* 2012). Because there is little evidence of AtlincRNA loci at the genomic level in poplar and grape (Liu *et al.* 2012), we restricted our search for sequence homologs to more recently diverged taxa. Similar sequences were identified using a reciprocal BLASTN approach (Johnson *et al.* 2008), sampling from the genomes of 10 Brassicaceae species plus *T. hassleriana* (Cleomaceae) (Figure 1A). In each of the plant genomes examined, we determined that an E-value cutoff of 1e–20 recovered similar sequences that were most often syntenic and returned the AtlincRNA query in reciprocal BLASTN searches. Herein, we refer to all loci that meet these three criteria as homologous. We then asked whether this E-value cutoff returned homologous sequences from other well-characterized lncRNA datasets as a means of further validating its use to recover homologous sequences in Brassicaceae. Using a random set of 10,000 human lncRNAs as query, we searched the *Pongo abelii* (orangutan) and *Mus musculus* (mouse) genomes, and identified homologous loci for 96% and 20% of the human lncRNAs in orangutan and mouse, respectively (Figure 2A), which is similar to previously reported percentages of 81% and 19%, based on genomic and transcriptomic approaches (Necsulea *et al.* 2014).

The Brassicaceae taxa sampled span a range of divergence dates with *A. thaliana* of ∼13 Mya to ∼65 Mya (Beilstein *et al.* 2010). Using a set of 5362 unique AtlincRNAs as query in a BLAST of Brassicaceae genomes (Figure 1, A and B), we found that the percentage of AtlincRNAs for which sequence homologs could be identified decreased as divergence date (phylogenetic distance) increased: *e.g.*, 23% for *Capsella rubella* (1233/5362; ∼18 Mya), 19% in *S. parvula* (1057/5362; ∼42 Mya), and 3% in *Aethionema arabicum* (186/5362; ∼54 Mya) (Figure 2B). Species of equal phylogenetic distance to *A. thaliana* differed in the number of AtlncRNA homologs recovered. For example, all lineage II species diverged from Arabidopsis ∼42 Mya, but we recovered homologs for only 16% of AtlncRNAs in *Brassica rapa*, whereas *S. parvula* and *E. salsugineum* harbored 19% and 20%, respectively (green bars, Figure 2B; *P*-value < 0.001). Using the same BLASTN parameters as those in our search for lincRNA homologs, sequence homologs were identified in the *A. arabicum* genome for 67% of a set of ∼10,038 *A. thaliana* protein-coding genes, and for 1% of 14,426 intergenic regions (blue and yellow bars, Figure 2B). Thus, AtlincRNA loci are conserved at an intermediate level in comparison to protein-coding genes and intergenic regions.

### Around 22% of AtlincRNA loci were present in the common ancestor of lineage I and II species ∼42 Mya

Following homolog identification in Brassicaceae genomes, we clustered the reciprocal BLASTN results from each pairwise AtlincRNA query into sequence families (Figure 1B). Each family contained the original query AtlincRNA, as well as homologous sequences from each subject genome that matched our criteria of synteny, reciprocity, and sequence similarity (E-value cutoff = 1e–20). From these families, we developed a more rarefied dataset of families composed of sequence homologs from a minimum of four species distributed between lineages I and II (Figure 1C). For example, in addition to the AtlincRNA query, all families were required to include sequences representing three other species, at least one of which had to be *B. rapa*, *B. oleraceae*, *S. parvula*, or *E. salsugineum* (representing Lineage II). Within the AtlincRNA dataset, 1271 loci (23%) met this criterion, and were grouped into unique families (Figure 1C and File S1). We did not permit returned homologs to be included in > 1 lincRNA family, and thus all families are unique. We refer to these families as conserved since these loci emerged, at minimum, in the most recent common ancestor of the two lineages ∼42 Mya (Beilstein *et al.* 2010).

We hypothesized that the observed sequence conservation of some AtlincRNA families could be due to the presence of short ORFs (< 100 aa), violating an important condition of inclusion as a putative lincRNA locus. To address this concern, these conserved families were screened for protein-coding potential via RNAcode (Washietl *et al.* 2011), and similarity to known noncoding RNAs, using the rFAM database (Nawrocki *et al.* 2015). RNAcode analyzes multi-sequence alignments for nucleotide substitutions or frameshifts that would maintain an ORF across multiple species. We found statistically significant evidence for a conserved ORF (*P*-value < 0.001; RNAcode) in 90 AtlincRNAs (Figure 1C). These families were excluded from further analysis but are listed in File S1. Moreover, we found 42 (3.5%) of the conserved set of AtlincRNA families contained sequences with significant similarity to a known noncoding RNA (*i.e.*, spliceosomal and snoRNAs; Figure 1C; listed in File S1). Indeed, 14 of the 51 AtlincRNAs with a sequence homolog in *T. hassleriana*, and nine of 17 AtlincRNAs with a sequence homolog in *Carica papaya* contain known noncoding RNA elements, explaining much of the sequence conservation seen in these more divergent genomes. In total, we identified 1180 (22%) conserved AtlincRNAs, for which sequence conservation was independent of coding potential (File S1). Of the original query AtlincRNAs used to build these conserved families, 93 have homologous sequences in all the Brassicaceae genomes we tested, and therefore represent an even more conserved dataset (File S1). In sum, we define here a class of conserved genomic regions that have been annotated as lincRNAs in *A. thaliana*. Whether these loci are conserved due to lincRNA function remains an open question.

### Overlap with CNS, transcription levels, and stress-responsiveness all correlate positively with conservation of AtlincRNA loci

We next attempted to understand factors influencing sequence conservation within the class of conserved AtlincRNA families. In general, protein-coding genes are more conserved than lincRNAs, both at the sequence level, and in regard to synteny (Goodstadt and Ponting 2006). However, intergenic regions can harbor important regulatory elements for protein-coding genes, and therefore display evidence of selective constraint. A recent comparative genomic analysis of sites under selection in Brassicaceae demonstrated that the percentage of sites in a genome under selection increases with proximity to the translation start site of the nearest protein-coding gene (Haudry *et al.* 2013). To address whether proximity of an AtlincRNA locus to a protein-coding gene might explain conservation of the latter, we tested for a positive correlation between proximity to a protein-coding gene, both up and downstream and on either strand, and sequence conservation (identification of a sequence homolog) for the AtlincRNA dataset. We detected no significant correlation between conservation of an AtlincRNA locus and its proximity to a known gene (Figure S1).

**Figure 3:**
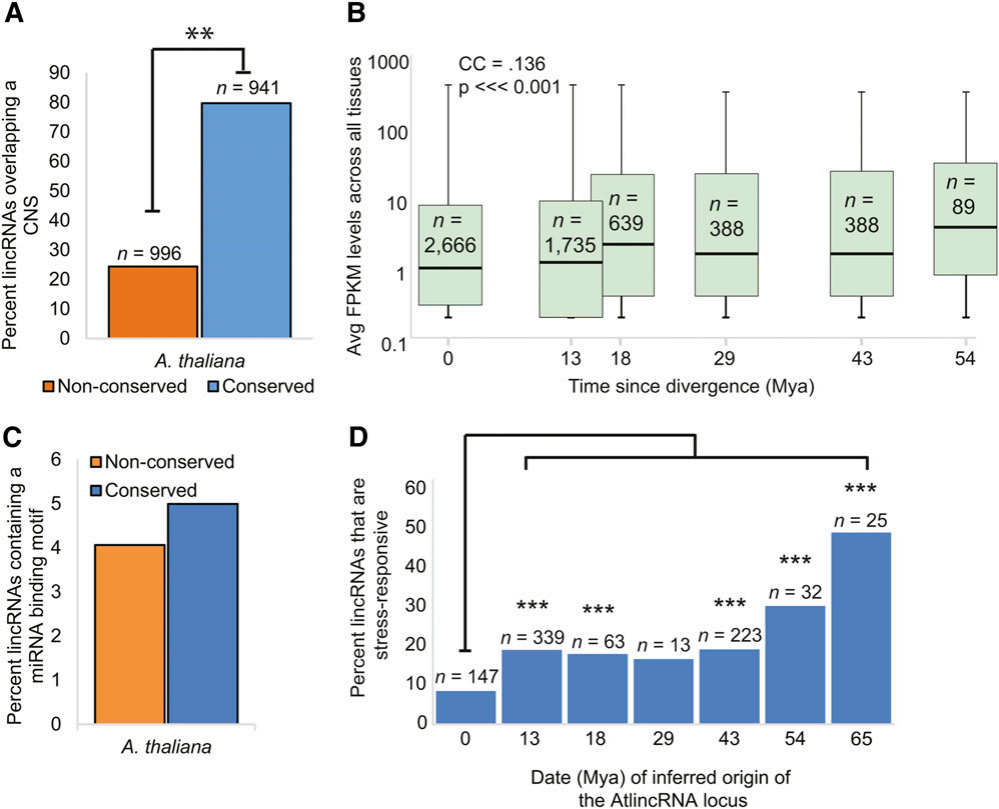
Features enriched in conserved AtlincRNAs. (A) Percent of AtlincRNA loci overlapping with conserved noncoding sequence defined by Haudry *et al.* (2013). Conserved AtlincRNA loci are defined by having sequence homologs in ≥ four species, with at least one species in the opposite lineage (*i.e.*, Lineage II). Nonconserved AtlincRNAs are those with < four sequence homologs. ** *P*-value < 0.001. (B) Box and whiskers plot of expression values for AtlincRNA families with homologous loci identified for increasingly divergent species. Expression is denoted as the average FPKM (fragment per kilobase of exon per million fragments mapped) values across four different tissues along a logarithmic scale [flowers, leaves, siliques, root; values from Liu *et al.* (2012)]. Transcription data were available for 2666 AtlincRNAs. The number of families with representatives at each divergence time-point is listed. Divergence times correspond to those shown in Figure 1A. A Pearson’s Correlation Coefficient was calculated (CC, top left). A linear regression analysis was performed to determine the statistical significance of this coefficient. (C) Percent of all nonconserved (orange) or conserved AtlincRNA (blue) families with miRNA binding motifs. (D) Percent of stress-responsive AtlincRNAs out of total number of AtlincRNAs conserved to each node (nodes indicates by divergence dates shown along *x*-axis). Actual number of stress-responsive AtlincRNAs shown above each bar. Where shown, *** indicates *P*-value < 0.0001 relative to the *A. thaliana*-specific lincRNAs (node 1).

Haudry *et al.* (2013) further identified a suite of 90,104 *A. thaliana* noncoding genomic regions (conserved noncoding sequences, or CNS) that showed a reduced substitution rate over contiguous regions. CNS have been identified in a variety of eukaryotes and are believed to be broadly important for gene regulation (Freeling and Subramaniam 2009; Adrian *et al.* 2010). In *A. thaliana*, these elements are typically short (on average 36 bp in length) and predominantly reside adjacent to (within 500 bp), or within (*i.e.*, untranslated regions and introns) genes. However, a subset (22%) of the identified CNS reside in intergenic space. Thus, we searched for overlap between these previously defined CNS and AtlincRNA loci. We detected a significant enrichment in overlap between intergenic CNS and the conserved AtlincRNA dataset (941/1180, or 80%) *vs.* the nonconserved AtlincRNA dataset (996/4082, or 24%; *P*-value < 0.001; Figure 3A). In sum, the presence of a CNS, but not proximity to a protein-coding gene, strongly correlates with genomic conservation of AtlincRNA loci.

Given the positive correlation between conservation of lincRNA loci and their expression and structure in vertebrates (Necsulea *et al.* 2014; Managadze *et al.* 2011), we tested if this paradigm also characterized genomically conserved AtlincRNAs. We inferred the most recent common ancestor in which the AtlincRNA locus was present based on our genomic comparisons. We used the divergence date of nodes in the tree to ask whether the expression level (FPKM) or structural complexity (Minimum Free Energy, MFE) of the AtlincRNA correlates with age of its emergence within the family (*i.e.*, most recent common ancestor [node] where the locus was likely present) (Figure 3B). We found that AtlincRNAs with an orthologous locus detectable in *A. arabicum*, and thus for which the ancestor of all extant Brassicaceae is inferred to have had a copy (54 Mya), were on average expressed at a higher level in *A. thaliana* than the average value for the population of *A. thaliana*-specific lincRNAs (Figure 3B). In fact, expression in *A. thaliana* is positively correlated with sequence conservation across Brassicaceae (Correlation Coefficient of 0.136, *P*-value < 0.0001). In contrast, we found no correlation between age of emergence of an AtlincRNA locus and MFE, as determined by RNAfold (Lorenz *et al.* 2011) (Figure S2).

Another possible explanation for sequence conservation is conservation of function. While it is difficult to infer function of a lincRNA from sequence alone, there are categories that are more amenable to functional prediction, such as natural antisense transcripts and microRNA sponges. The lincRNAs in our dataset do not overlap known genes (in either direction), and therefore cannot be antisense transcriptional regulators. However, given the recent reports of lncRNAs acting as molecular sponges of miRNAs (Kretz *et al.* 2012; Hansen *et al.* 2013), we assessed the potential of conserved AtlincRNA loci to bind miRNAs. We searched for miRNA binding sites using the Brassicaceae miRNA dataset in psRNATarget (Dai and Zhao 2011). AtlincRNA families with putative miRNA binding sites make up 4% of the overall lincRNA population. We observed a modest enrichment in miRNA binding sites in the conserved AtlincRNA dataset (5% *vs.* 4%; Figure 3C; a list of lincRNAs with miRNA binding sites is provided in File S1). Interestingly, in 9 out of the 59 AtlincRNAs that harbored miRNA binding sites, the sequence of the motif was conserved at the same locus in all Brassicaceae, potentially representing a deeply conserved lincRNA regulatory pathway.

Finally, we asked if the conserved AtlincRNAs were over-represented in the stress-responsive lincRNA dataset produced by Liu *et al.* (2012). In the dataset of 5270 AtlincRNAs that we examined, 969 were differentially expressed in response to at least one of four environmental stresses (abscisic acid, cold, drought, and salt) (Liu *et al.* 2012). We determined whether the proportion of stress-responsive lincRNAs increased with the inferred age of emergence. More specifically, for all 5270 AtlincRNAs, we determined the species with the deepest coalescent point in the organismal tree from which a sequence homolog was retrieved, and then, for each point, we calculated the percentage of lincRNAs classified as stress responsive. For example, 1736 lincRNAs are *A. thaliana*-specific, and 147 of these (8.5%) were stress-responsive (Figure 3D). For the 1785 lincRNAs that coalesce at the node uniting *A. thaliana* and *A. lyrata*, 339 were stress-responsive (19.0%, *P*-value < 0.0001; Fisher’s exact test relative to stress responsive *A. thaliana*-specific lincRNAs); 106 lincRNAs coalesce at the node uniting *A. thaliana* and *A. arabicum*, 32 stress responsive (30.0%, *P*-value < 0.0001). And, for the 25 lincRNAs that coalesce at the node uniting *A. thaliana* and *T. hassleriana*, 12 were stress-responsive (49.0%, *P*-value < 0.0001) (Figure 3D). Thus, conservation of an AtlincRNA locus correlates with its propensity to be differentially regulated in response to environmental stress in *A. thaliana*. A list of these lincRNAs is provided in File S1.

### Gene/whole genome duplication, but not transposable elements, influence lincRNA diversification

**Figure 4:**
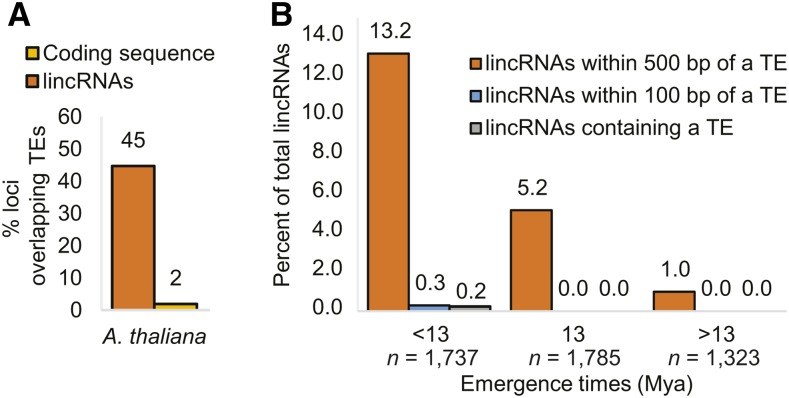
Transposable element (TE) content in AtlincRNAs. (A) Percent of lincRNAs and coding sequences from *A. thaliana* that overlap with ≥ 10 bp of a TE as determined by RepeatMasker. Actual percent shown above each bar. (B) Connection between TEs and AtlincRNA emergence. AtlincRNAs were binned based on when they are believed to have emerged [shown on *x*-axis in millions of years ago (Mya)]. TE content, either within or adjacent to the lincRNA, was determined for AtlincRNAs within each bin.

We next identified genomic factors that might be driving emergence or loss of AtlincRNA homologs in Brassicaceae genomes. Recent findings in vertebrates suggest a role for TEs in lncRNA diversification (Kapusta *et al.* 2013). The AtlincRNA dataset of Liu *et al.* (2012) excluded sequences with fragments of TEs, precluding comparison with results in vertebrates. To remedy this issue, and to explore the potential role of TEs in lincRNA diversification, we reanalyzed the AtlincRNA dataset, including the intergenic transcripts previously shown to contain repetitive elements, using the same filtering parameters used in the vertebrate study (Kapusta *et al.* 2013). This yielded ∼12,000 putative AtlincRNAs, 45% of which contained at least 10 nt of TE DNA (Figure 4A and File S2). Similar to vertebrates, AtlincRNA loci contained significantly more TE content than protein-coding loci (*P* < 0.01; Fisher’s exact test). However, the percentage of AtlincRNA loci containing at least 10 nt of a TE was significantly less than that reported for vertebrate lncRNAs (*P* < 0.01; Fisher’s exact test) (Kapusta *et al.* 2013). We also asked if AtlincRNA emergence correlated with the presence of a TE, either within the lincRNA, or in the region upstream or downstream of the lincRNA (Figure 4B). A small percentage (0.2%) of the species-specific AtlincRNA loci (*i.e.*, appear to have emerged since *A. thaliana* and *A. lyrata* diverged ∼13 Mya) contained at least 10 nt of TE DNA. A larger percentage (13.2%) of the species-specific AtlincRNAs were within 500 bp of TE DNA. None of the conserved AtlincRNAs contained, nor were they within 100 bp of, any TEs. Thus, for the loci encoding AtlincRNAs in Brassicaceae, we find little evidence to indicate that transposable element activity promotes the emergence of new lincRNAs in the genome. However, as seen in vertebrates, adjacent TEs may be driving expression of these species-specific lincRNA loci (Kapusta *et al.* 2013; Kelley *et al.* 2014).

**Figure 5:**
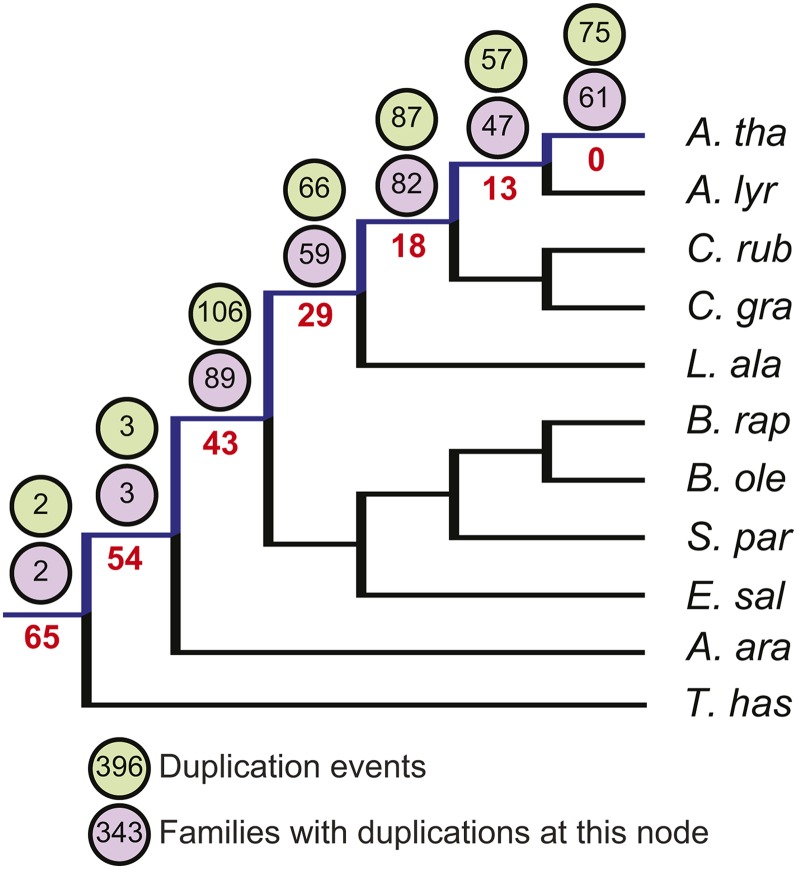
Inferred timing of duplication and duplication dependence in the conserved AtlincRNA families. Timing of duplications within *A. thaliana* conserved lincRNA families. The data represent duplications that occur along the backbone leading to the AtlincRNA (blue bar). Duplications are shown per node, with approximate divergence times (Mya) shown in red. The number of duplication events per node are shown in the green circles. The number of AtlincRNA families with duplications per node are shown in purple circles. Some families contained a duplication at multiple nodes and therefore were counted multiple times. Overall, 296 AtlincRNA loci showed evidence of a duplication event at least once, but in some cases multiple times. The total number of duplications are shown below.

Given the prevalence of gene duplicates in plant genomes, and in Brassicaceae specifically (Koenig and Weigel 2015), we asked whether duplication events, either in the form of WGD or local duplication, might be a mechanism for lincRNA emergence. To investigate the impact of duplication events on lincRNA evolution, we inferred the most likely gene tree, and estimated branch support using maximum likelihood bootstrap for each of the 1180 conserved AtlincRNA families, and then employed Notung (2.0) (Durand *et al.* 2006) to determine the duplication history. Notung reconciles topological incongruence between gene trees and the accepted organismal tree, using incongruence to infer duplications and losses. We analyzed duplication in all conserved AtlincRNA families but omitted highly duplicated families (> 3 duplication events along the backbone of the tree) from downstream analysis. Of the remaining 1005 conserved families, 296 (29%) showed evidence of at least one, but sometimes multiple duplication events along the branch leading to *A. thaliana* (blue line, Figure 5; lincRNA IDs listed in File S2), indicating that numerous AtlincRNAs are likely the product of relatively recent duplication events. In general, duplications were relatively evenly distributed along the backbone nodes leading to *A. thaliana*, although 106 families (26%) experienced a duplication event along the branch uniting lineage I and lineage II (Figure 5). Due to the low number of identifiable homologs in *A. arabicum* and *T. hassleriana*, we recovered only a few duplication events that trace back to the deepest nodes in our tree. It should be noted that no duplication event coincided with the insertion of a TE, either within or adjacent to the lincRNA locus. In sum, gene duplication appears to have played a role in the evolution of approximately one-third of conserved AtlincRNAs. Moreover, the gene duplication events driving lincRNA evolution do not appear to be due to the activity of transposable elements.

**Figure 6:**
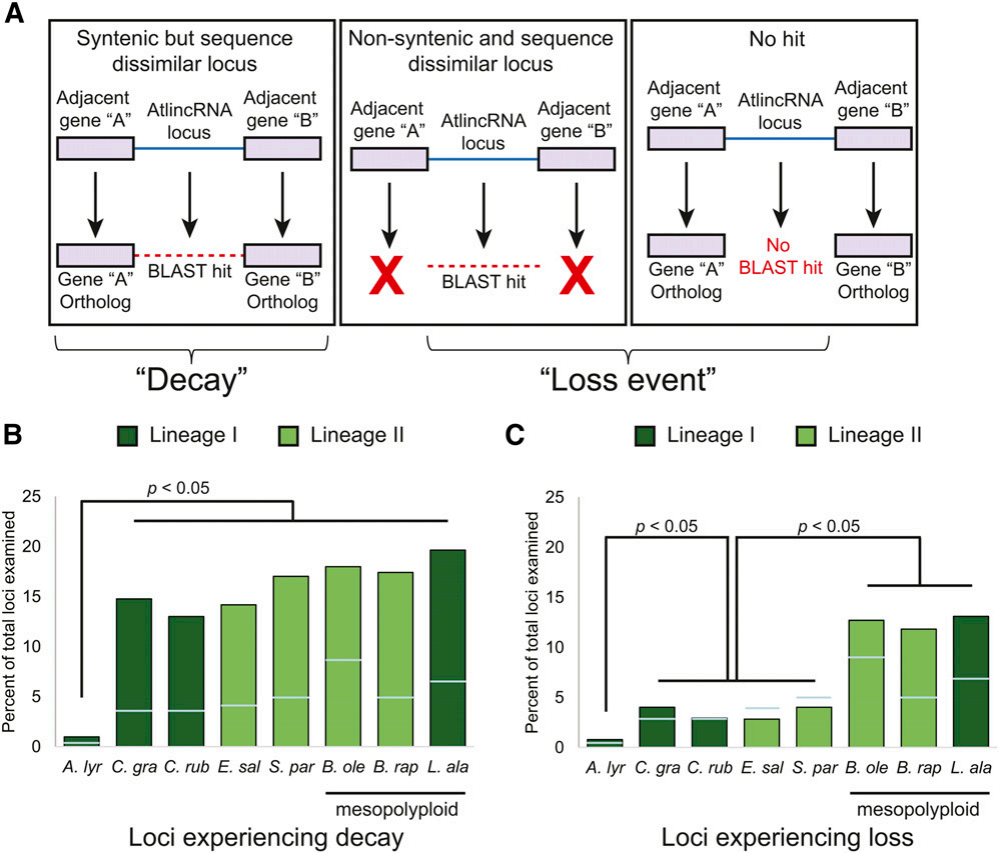
Sequence loss and decay events in the conserved AtlincRNA families. (A) Strategy for inferring sequence decay or loss for the absent loci in the conserved AtlincRNA families using a less stringent BLASTN cutoff (1e–5) and synteny. (B) Bar graph of the percent (out of total 1023) of lincRNA loci experiencing sequence decay in the species listed. Pairwise comparisons of the proportion of lost or decayed loci were performed between all species using a score test for a difference of binomial proportions. Species that, after a Bonferroni correction, were not significantly different from one another were grouped. (C) Bar graph of the percent (out of total 1023) of lincRNA loci experiencing loss in the species listed. Raw numbers are shown in File S2. Light blue bars depict the level of loss and decay observed for protein-coding loci.

Conserved AtlincRNA families are required to include a representative from both lineages I and II, indicating that the locus was present in the common ancestor of species in these groups (red asterisk, Figure 1C). Therefore, the lack of an AtlincRNA-like homolog from a species in these lineages suggests that either: 1) the locus was purged from the genome, or 2) it has accumulated sufficient nucleotide divergence to prevent identification at our BLASTN cutoff value. We refer to these alternatives as lincRNA locus loss or decay, respectively. To infer rates of loss and decay, we repeated the reciprocal BLASTN search using a less stringent E-value cutoff (1e–5) (Figure 6A). For sequence variable loci (*i.e.*, recovered between 1e–20 and 1e–5), we determined whether they shared synteny with the AtlincRNA query, and, if so, classified them as decay events in that species. Alternatively, if we failed to recover additional BLASTN hits at lower stringency, or the recovered sequences were in different genomic locations than the AtlincRNA query, they were classified as loss events (Figure 6A).

We identified the number of loci that were lost or decayed and performed pairwise comparisons between all species. Based on these comparisons, we identified three distinct groups of species (Figure 6, B and C). The percent of decayed loci was similar for the group containing *S. parvula*, *B. oleracea*, *B. rapa*, and *L. alabamica* (17.0–19.6%), while a lower percent of decay characterized the group containing *Capsella grandiflora*, *C. rubella*, and *E. salsugineum* (13.0–14.8%; Figure 6B and File S2). The percent of decayed loci was significantly different between these two groups (*P* < 0.01 based on pairwise comparisons using a Bonferroni multiple comparison correction; see File S2). *A. lyrata* experienced significantly less decay than either of these two groups (1%; *P* < 0.0001; Bonferroni multiple comparison correction). The groupings identified by pairwise comparisons for loci experiencing loss were composed of different species. The pairwise differences between the mesopolyploids *B. oleracea*, *B. rapa*, and *L. alabamica* were insignificant and ranged from 11.8% to 13.0% (Figure 6C). In relation to each other, similar percentages of loss (2.8–4.0%) were detected in the nonpolyploid species *C. grandiflora*, *C. rubella*, *E. salsugineum*, and *S. parvula*. The percent of lost loci was significantly different between these two groups (*P* < 0.0001; Bonferroni multiple comparison correction). Loss of lincRNA loci was rare in *A. lyrata* (0.8%). Interestingly, the species for which we observed a greater than expected increase in lincRNA loss have experienced a recent WGD (mesopolyploidization event) (Kagale *et al.* 2014), suggesting the two may be correlated.

We also examined a randomized set of 10,611 *A. thaliana* protein-coding loci for loss and decay. We detected significantly elevated levels of loss and decay in *B. oleraceae* compared with other species, while *A. lyrata* showed significantly lower levels (File S2). Thus, for protein-coding genes, we did not detect groups of species with similar levels of loss and decay that correlated with WGD or phylogenetic position.

## Discussion

### A subset of AtlincRNAs are conserved across Brassicaceae and may be cis-regulatory RNAs

The evolution of AtlincRNA loci is broadly similar to that seen in vertebrate systems, wherein sequence conservation is inversely proportional to timing of divergence. The percent of lncRNAs found to be homologous between humans and mice, which diverged ∼90 Mya, ranges from 19% to 38% (Necsulea *et al.* 2014; Washietl *et al.* 2014; Hezroni *et al.* 2015). Of the human lncRNA dataset used by Necsulea *et al.* (2014), ∼3% can be identified in chicken, which diverged from humans > 300 Mya. In our analysis, a large percentage of the AtlincRNAs appear to be either species- or genus-specific, thus explaining previous reports on the extremely low (< 1%) recovery of sequence homologs between more distantly related species [*i.e.*, *Arabidopsis* and poplar (Shuai *et al.* 2014; Liu *et al.* 2012)]. Despite this variation, we identified a group of 1180 lincRNA loci with sequence homologs in both lineage I and II of the family. Within this conserved set, sequence homologs were detected in all tested Brassicaceae genomes for 93 AtlincRNAs, dating the origin of these loci to at least 54 Mya. Thus, our comparative genomic analysis serves as an additional filter in the identification of conserved AtlincRNA loci. The implications of this sequence conservation are unclear, but could be due to conservation of lincRNA function.

We identified several factors that may best explain the genomic conservation we observed for 22% of the AtlincRNA loci. Features that were enriched within the conserved AtlincRNA dataset include higher overall expression, stress-responsiveness, and overlap with previously identified intergenic conserved noncoding sequences (CNS). The propensity of more deeply conserved AtlincRNAs to overlap with CNS is particularly interesting because these DNA elements are predicted to be *cis*-acting transcriptional regulators (Freeling and Subramaniam 2009). Examples of this regulation include a CNS referred to as *Vgt1* that is associated with flowering time in the grasses (Salvi *et al.* 2007). Here we note overlap between the AtlincRNA dataset of Liu *et al.* (2012) with the CNS from Haudry *et al.* (2013), to our knowledge providing significant evidence of CNS transcription for the first time. Given the size difference between CNS (∼36 bp) and lincRNAs (>200 nts), overlap with a CNS is not enough to explain the retention we see in the more conserved AtlincRNA dataset, suggesting sequence conservation is driven by additional factors. More importantly, transcription of CNS as lincRNAs suggests that these regions might regulate gene expression at the RNA level as well.

Vertebrate enhancer regions are important *cis*-regulatory elements that show signatures of selection and, in some cases, control cell- and tissue-specific expression profiles. Some have argued that plant CNSs are functional analogs of vertebrate enhancers (Freeling and Subramaniam 2009; Pennacchio *et al.* 2007; Lam *et al.* 2014). Large-scale transcriptomic analyses indicate that many of these enhancer regions are transcriptionally active (Djebali *et al.* 2012). When transcribed, they are termed enhancer RNAs (eRNAs), and data support a model in which the presence of the eRNA, and not just transcription of the enhancer region, regulates expression of adjacent genes (Lam *et al.* 2013). While evidence of transcription of Arabidopsis CNS is not sufficient to demonstrate that CNS-overlapping lincRNAs are enhancer RNAs, this result suggests that further study is warranted.

### Genome dynamics are driving diversification of lincRNA-encoding loci

The burgeoning interest in lncRNAs and the observation that a large set of them are species specific, have propelled studies focused on identifying factors influencing their diversification. Transposable elements are implicated in diversification of lincRNA populations in vertebrates (Kapusta *et al.* 2013). Similar to vertebrate analyses, AtlincRNAs contain more TE content than protein-coding genes. Nevertheless, species-specific AtlincRNAs were no more likely to contain a TE than AtlincRNAs with sequence homologs in other Brassicaceae, suggesting that transposable element activity is not driving species-specific AtlincRNA emergence. However, we noticed an increase in the number of species-specific lincRNA loci within 500 bp of a TE compared with AtlincRNA loci for which similar sequences were identified in the genomes of other Brassicaceae. Thus, it may be that TEs are acting as *cis*-regulatory elements, facilitating transcription of these lincRNA loci, similar to observations in humans and other vertebrates (Kelley *et al.* 2014; Kapusta *et al.* 2013). Brassicaceae genomes are relatively depauperate in TEs when compared with genomes in grasses or other plant families (Murat *et al.* 2012), whose TE content is more similar to that in vertebrates. Hence, it is possible that the lack of influence exerted by TEs we observed in Brassicaceae may not be representative of other groups of plants. Add to this the observation that lincRNAs in grasses are less conserved genomically than are those in Brassicaceae (Li *et al.* 2014; Xin Qi *et al.* 2013), and a reasonable hypothesis moving forward is that lincRNA diversification driven by TEs depends on their abundance and level of activity in the genome.

WGD, given its prevalence in plants, presents another likely mechanism for emergence of lincRNAs (Husband *et al.* 2013; Moghe and Shiu 2014). All Brassicaceae share a WGD termed the α duplication, and, if this event precipitated the emergence of lincRNAs in the group, it could explain the inability to find AtlincRNA sequence homologs in *T. hassleriana* (Cleomaceae) and *C. papaya* (Caricaceae), whose divergences predate the WGD (Beilstein *et al.* 2010; Cheng *et al.* 2013; Koenig and Weigel 2015). However, our analyses of gene duplication did not recover an overrepresentation of lncRNAs with duplications along the same branch that the α WGD occurred. Instead, lincRNA duplication events associated with AtlincRNAs were fairly evenly distributed along the backbone leading to *A. thaliana*. The lack of a correlation between known WGD and lincRNA emergence implies that the duplications we detected are local rather than global events. In contrast to emergence, we found that WGD events correlate with an accelerated loss of lincRNA loci. This is consistent with observations for *B. rapa* protein-coding loci that indicate deletions, and not point mutations, make up the bulk of the gene fractionation that has occurred postpolyploidization (Tang *et al.* 2012). In sum, our data suggest that recent WGD may contribute to variability in the persistence of putative lincRNAs among species by increasing the rate of their deletion, likely due to fractionation post polyploidy. Thus, the α WGD event that defines Brassicaceae may have led to a dramatic decline in the ancestral lincRNA population, resulting in very few lincRNAs with conserved loci throughout the family (Nelson and Shippen 2015).

A significant caveat to these analyses is that conservation of a lincRNA-encoding locus does not imply expression, and thus it is not clear if expression is conserved across the family. However, even with these limitations, comparative genomic approaches can still be informative in systems with minimal transcriptomic data. Due to their above average sequence conservation, the conserved lincRNA dataset described here represents an excellent starting point for functional analysis. For example, several of the conserved and stress-responsive AtlincRNAs we identify here were recently shown to be protein-bound and nuclear localized, providing further evidence that signatures of conservation may underlie conservation of function across Brassicaceae for well conserved AtlincRNAs (Gosai *et al.* 2015).

## Supplementary Material

Supplemental Material
