# Supplementary material for: A Genomic Analysis of Factors Driving lincRNA Diversification: Lessons from Plants
Source: G3 (Bethesda). 2016 Jul 15;6(9):2881–91. doi: 10.1534/g3.116.030338 (PMC5015945; doi:10.1534/g3.116.030338)
Supplement: Supplemental Material [file supp_6_9_2881__index.html]

A Genomic Analysis of Factors Driving lincRNA Diversification: Lessons from Plants — Supplemental Material 

# A Genomic Analysis of Factors Driving lincRNA Diversification: Lessons from Plants

## Supplemental Material for Nelson, *et al*, 2016

**Files in this Data Supplement:**

- Figure S1 - Proximity of AtlincRNAs to nearest protein-coding gene. AtlincRNAs were binned into different groups based on their estimated date of emergence. Then the average distance (in bp) from the end of a lincRNA to the nearest protein-coding gene was calculated for each group. (.pdf, 275 KB)
- Figure S2 - Test for a correlation between lincRNA structure and conservation at the genomic level. (.pdf, 249 KB)
- File S1 - Output of comparative genomic analysis for AtlincRNAs, including rFAM and miRNA analysis. (.xlsx, 410 KB)
- File S2 - Overview of factors influencing evolution of AtlincRNA loci. (.xlsx, 84 KB)
