# Supplementary material for: A Genomic Analysis of Factors Driving lincRNA Diversification: Lessons from Plants
Source: G3 (Bethesda). 2016 Jul 15;6(9):2881–91. doi: 10.1534/g3.116.030338 (PMC5015945; doi:10.1534/g3.116.030338)

Nelson et al, Supplemental Figure 3

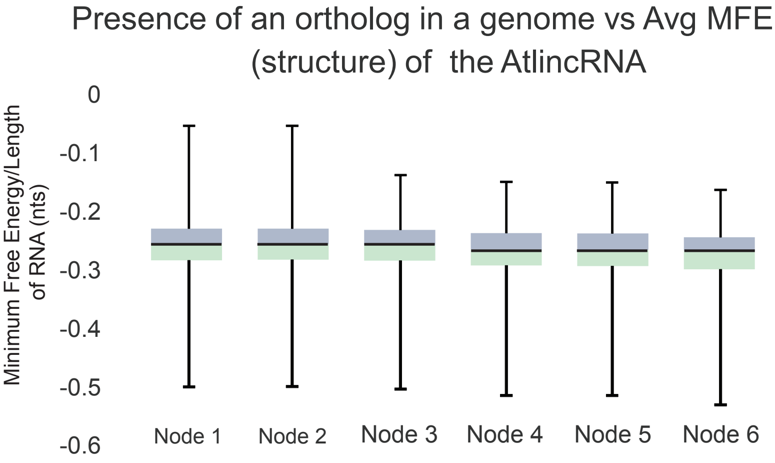

Supplement: Supplemental Material [file supp_g3.116.030338_FigureS2.pdf]
